# Editorial: Stromal and immune microenvironment in breast cancer metastasis

**DOI:** 10.3389/fimmu.2022.1104362

**Published:** 2022-12-06

**Authors:** Xin Lu, Xia Liu, Toni Celià-Terrassa, Guangwen Ren

**Affiliations:** ^1^ Department of Biological Sciences, Boler-Parseghian Center for Rare and Neglected Diseases, Harper Cancer Research Institute, University of Notre Dame, Notre Dame, IN, United States; ^2^ Tumor Microenvironment and Metastasis Program, Indiana University Melvin and Bren Simon Comprehensive Cancer Center, Indianapolis, IN, United States; ^3^ Department of Toxicology and Cancer Biology, Markey Cancer Center, College of Medicine, University of Kentucky, Lexington, KY, United States; ^4^ Cancer Research Program, Hospital del Mar Medical Research Institute, Barcelona, Spain; ^5^ The Jackson Laboratory, Bar Harbor, ME, United States

**Keywords:** breast cancer metastasis, chronic inflammation, eosinophil, tumor necrosis factor, cuproptosis, single-cell multiomics

Breast cancer is the most common cancer type in women and breast cancer-related deaths are mainly due to the metastases of tumors to the distant vital organs including the lung, liver, bone and brain. In the past three decades, significant advances in breast cancer metastasis research have revealed intricate interactions between tumor cells and the tissue microenvironments that are essential for the development of organ metastatic lesions. Within both primary tumor and distant organ microenvironments, immune infiltrates and stromal cells are the major components. Through chemical or physical interactions with breast cancer cells, these non-neoplastic cells play instrumental roles in the progression and metastasis of breast cancer. Recent technological advances, particularly the high throughput sequencing and proteomics, make it possible to reveal the heterogeneity and complex interactions of stromal cells, immune infiltrates and tumor cells at the single cell and spatial levels. This Frontiers Research Topic entitled “Stromal and Immune Microenvironment in Breast Cancer Metastasis” has collated 5 contributions from experts who are exploring the cancer-immune crosstalk and its role in the disease progression and prognosis of metastatic breast cancer.

Eosinophils as a minor population of granulocytes that infiltrate breast cancer have a poorly understood function in cancer progression, despite their known role in allergy and Th2 immunity. Cederberg et al. explored the role of eosinophils in breast cancer metastasis to lung by employing transgenic mouse models with hypereosinophilia (IL5Tg mice) and hypoeosinophilia (ΔdblGATA mice), as well as antibody-mediated depletion of eosinophils (Cederberg et al., 2022). These transgenic mice were used as the host to examine the growth of murine syngeneic mammary tumor cells EO771 in the lungs. Pulmonary metastasis formation was significantly reduced in IL5Tg mice compared to wild-type mice or ΔdblGATA mice. Concordantly, eosinophil depletion accelerated lung metastasis. Mechanistically, lung eosinophils were activated during metastasis development, and expressed activation markers and released eosinophil peroxidase to kill tumor cells. These results establish a metastasis-suppressor role of eosinophils in breast cancer, illuminating a promising therapy for breast cancer lung metastasis by triggering local eosinophil degranulation.

Chronic inflammation is a hallmark of solid tumors. In the context of breast cancer, chronic inflammation appears to be an important risk factor and a potential promoter for the disease progression. Among the factors that regulate tissue inflammation, tumor necrosis factor family of ligands (TNFSFs) and receptors (TNFRSFs) have prominent roles. Ekstrand et al. explored the association between the levels of TNFSFs/TNFRSFs and breast tissue density or local breast estradiol levels with a cohort of 73 women including 12 with breast cancer, 42 healthy postmenopausal women with different breast densities, and 19 healthy premenopausal women. Among the 23 members of the TNFSF/TNFRSF families measured by microdialysis and proximity extension assay, the majority of TNFSF and TNFRSF proteins were significantly upregulated in breast cancer compared with normal adjacent breast tissue. In healthy postmenopausal women, dense breast tissue exhibited a similar dysregulation pattern as breast cancer, when compared with the non-dense breasts. Furthermore, estradiol correlated with most of the TNFSF/TNFRSF members, which was further supported in ER+ breast cancer mouse model. This study reveals the potential of the specific TNFSF/TNFRSF family members as biomarkers for breast cancer prevention (especially for postmenopausal women with dense breasts) and as therapeutic targets for estrogen-dependent breast cancer.

Recently, non-apoptotic regulated cell death mechanisms are among the most exciting fronts of cell biology research. Among these mechanisms, cuproptosis is copper-dependent regulated cell death that relies on mitochondria respiration and exhibits emerging roles in tumor progression and immune response. Sha et al. performed a comprehensive bioinformatics analysis to explore the potential role of cuproptosis-related genes (CRGs) in the tumor microenvironment of triple-negative breast cancer. The TCGA dataset and three GEO datasets were analyzed systematically. Interestingly, two CRG clusters were identified that correlated well with the inflamed (hot) immune infiltration pattern and the immune-desert (cold) pattern of breast cancer. Whereas the high CRG scores were associated with the stromal activation and immunosuppression, the low CRG scores were associated with high tumor mutation burden, immune activation and better survival. Based on this study, specific hypotheses can be generated to interrogate the functions of the disparately scored CRG genes in breast cancer immunity and immunotherapy response.

Single-cell multiomics, especially single-cell RNA sequencing (scRNA-seq), is a transforming technology for biological research, including breast cancer research. Tan et al. reviewed recent literature that profiled breast cancer microenvironment with single-cell technologies (scRNA-seq, spatial transcriptomics, CyTOF) and revealed the vastly complex heterogeneity in the immune and non-immune compartments and their functional states. A comprehensive table was provided to summarize the major constitutional cell populations (CD4+ T cells, CD8+ T cells, B cells, macrophages, neutrophils, monocytes, cancer-associated fibroblasts), the refined cell subtypes and markers for each major population, and their functions in the regulation of tumor immunity. With the wealth of information reviewed and summarized in an accessible format, this review article provides an excellent resource for readers interested in exploring the breast cancer cellular heterogeneity in depth.

The crosstalk among different cell populations in either chemical signaling or mechanical form represents one of the most complex and fascinating aspects of the tumor microenvironment research. Deeper understanding of the tumor-immune and stroma-immune interactions may lead to novel therapeutic avenues to enhance the current treatment regimens especially immunotherapy. Tan et al. reviewed the recent literature on this subject in the context of breast cancer metastasis and categorized the findings to topics including invasion/intravasation, extravasation/colonization, pre-metastatic niche, survival in the vascular and lymphatic systems, and therapy resistance. This review provides a roadmap for researchers in the breast cancer metastasis field to grasp the cutting-edge findings on this subject and to identify the knowledge gaps for future studies.

Overall, these publications in the Research Topic are expected to advance our understanding of the microenvironmental regulation of breast cancer metastasis and potentially lead to exciting new therapeutic opportunities for patients with metastatic breast cancer.

